# Silicon enhances salinity tolerance in tomato (*Solanum lycopersicum* L.) through physiological, biochemical, and ionic regulation

**DOI:** 10.1186/s12870-026-09859-y

**Published:** 2026-09-09

**Authors:** Ibrahim Badawy, Heba I. Mohamed, Hossam Fouda, Mohamed E. Elnosary, Mahmoud R. Sofy

**Affiliations:** 1 Botany and Microbiology Department, Faculty of Science, Al-Azhar University, Cairo, Egypt; 2 Biological and Geological Sciences Department, Faculty of Education, Ain Shams University, Roxy, Cairo, 11341 Egypt

**Keywords:** Ascorbic acid, Glutathione, Oxidative stress, Antioxidant enzymes, Ion homeostasis, Photosynthesis, Proline

## Abstract

**Background:**

Soil salinization poses a significant threat to agricultural production, necessitating innovative agronomic strategies to mitigate its impact. Salinity stress is a critical abiotic factor that hampers tomato growth by causing ionic imbalances, oxidative damage, and disruptions in physiological systems. This study investigated the effect of sodium silicate (Na₂SiO₃) in reducing salt stress in tomato plants exposed to 75 and 150 mM NaCl.

**Results:**

The results indicated that salinity substantially decreased plant growth, the content of photosynthetic pigments, and the uptake of essential mineral nutrients. Concurrently, it heightened oxidative stress markers such as malondialdehyde (MDA), electrolyte leakage, hydrogen peroxide (H₂O₂), and hydroxyl radicals (•OH). Treatment with Na₂SiO₃, particularly at a concentration of 2 mM, significantly improved growth performance and photosynthetic pigment content. Silicon treatments enhanced the antioxidant defense mechanisms by increasing the activity of superoxide dismutase (SOD), catalase (CAT), ascorbate peroxidase (APX), glutathione reductase (GR), and peroxidase (POX). Additionally, levels of ascorbate (AsA) and glutathione (GSH) were raised, leading to reduced oxidative damage and stabilized cellular membranes. The application of silicon also improved ionic homeostasis by decreasing sodium ion (Na⁺) concentration while enhancing the absorption of nitrogen (N), phosphorus (P), potassium (K⁺), and silicon.

**Conclusions:**

In conclusion, the study demonstrated that silicon effectively alleviates salt stress in tomato plants by improving redox balance, enhancing antioxidant capacity, and regulating ion transport. The most effective concentration of Na₂SiO₃ was found to be 2 mM. The exogenous application of sodium silicate, especially at this concentration, helps to mitigate salinity-induced impairments in tomato plants by enhancing physiological performance, antioxidant defense, and ionic balance. This suggests its potential use as a sustainable agricultural strategy under salt-affected conditions.

## Introduction

 Rapid population increase and the continuous decline in arable land require sustainable approaches aimed at maintaining and improving the productivity of crops [1, 2]. In addition, the modern approach towards agriculture requires reaching two goals, namely, obtaining enough and high-quality products along with minimizing any environmental impacts that remain the main global issues [3–5]. Soil salinization has become one of the most significant limiting factors of the agricultural industry since it interferes with the physiological processes of the plants, limits the growth of plants, and causes osmotic stress and nutrient imbalance [6]. The issue of soil salinization currently concerns about 20% of the global cultivated soils, and the problem can become even more pressing because of climate change and irrigation [7]. Thus, the creation of new agricultural approaches and crop management techniques for enhancing the salt tolerance of plants becomes extremely relevant [8–10].

The use of silicon (Si) has received more interest as a beneficial element and biostimulant that could help enhance plant resistance against salt stress and other kinds of environmental stress [11–13]. The use of Si could help maintain water relationships in plants, keep ionic balance, reduce Na⁺ levels, and improve the ratio of K⁺ to Na⁺ under saline conditions. Si could also help maintain the efficiency of photosynthesis as well as protect antioxidant activity through enzyme activity such as SOD (superoxide dismutase), CAT (catalase), APX (ascorbate peroxidase), POX (peroxidase), and GR (glutathione reductase). This means that it would be able to control reactive oxygen species (ROS). In addition, the use of Si could help develop non-enzymatic antioxidants and increase the strength of leaves through silica deposition [14].

Tomato (*Solanum lycopersicum* L.) is one of the commercially and nutritionally valued vegetables grown globally [15]. Despite the presence of salt tolerance in tomato, it is a glycophyte plant species that suffers due to salinity stress by osmotic stress, ion toxicity, oxidative damage, and metabolic disturbances [16]. The use of silicon (Si) has been proposed as an effective means of enhancing the salt tolerance ability of plants by sustaining photosynthesis efficiency, inhibiting Na⁺ ions uptake, and antioxidant activity [17, 18]. In addition, Si has the potential of promoting root development and absorption of nutrients, hence ionic and nutritional balance [19]. Nevertheless, the effectiveness of Si application depends on its concentration and level of salinity, implying that the need for identifying appropriate concentrations of Si for tomato enhancement is necessary.

We hypothesized that foliar application of sodium silicate (Na₂SiO₃) would alleviate salt-induced damage by improving ionic homeostasis, reducing oxidative stress, enhancing antioxidant defense and secondary metabolism, and consequently improving tomato growth and physiological performance, with the magnitude of these responses varying according to the applied Si concentration. So, the current study was designed to determine the negative impacts of salinity stress caused by sodium in tomato plants regarding their growth, oxidative stress, secondary metabolism, and antioxidant enzyme systems, and to investigate the effect of silicon application on reducing sodium toxicity and enhancing stress tolerance in tomato plants.

## Materials and methods

### Plant material and experimental design

Tomato seeds (*Solanum lycopersicum* L., variety ‘Super Strain B’) was classified as a standard, moderately salt-tolerant genotype and were purchased from the Agricultural Research Center (ARC), Giza, Egypt. Seeds that were uniform in size and appearance and showed good health were chosen and surface-sterilized using 1% (v/v) sodium hypochlorite solution for 10 min and then washed thoroughly with distilled water. Seven-day-old seedlings were transferred into sterilized plastic pots (40 cm diameter) containing a soilless mixture of peat moss (33%), clay (30%), and sand (37%). Greenhouse experiments were carried out at a temperature of 27/21°C, a relative humidity of 53–57%, a photosynthetic photon flux density (PPFD) of 100–200 µmol m⁻²s⁻¹, and a CO₂ concentration of 300–410 µmol m⁻²s⁻¹.

The physicochemical characteristics of the growth medium were analyzed prior to transplanting. The substrate contained a pH of 7.15 and electrical conductivity (EC) of 1.32 dS m⁻¹. Among the main anions (meq L⁻¹) are SO₄²⁻ (5.42), Cl⁻ (4.15), and HCO₃⁻ (1.31). Among the main cations (meq L⁻¹), they are Ca²⁺ (4.10), Na⁺ (4.19), Mg²⁺ (1.32), and K⁺ (0.36). Seedlings were transplanted 15 days after sowing (DAS). Stress conditions were imposed using the NaCl solution at different concentrations (75 and 150 mM) twice daily for two days. Control plants were treated without NaCl solution. In order to prevent any osmotic shock due to Si application, a solution of sodium silicate (Na₂SiO₃; Merck KGaA, Darmstadt, Germany) was applied as a foliar spray in concentrations of 1, 2, and 3 mM on the 7th day after NaCl treatment (22 DAS). The second foliar spray of Si was applied 7 days later (29 DAS). Foliar spraying was done in the morning by means of a hand-held spray bottle with a calibrated pressure pump so that even droplet distribution was achieved on the upper and lower sides of the leaves, about 15–20 mL of solution per plant. The experiment was designed using a completely randomized design (CRD) with twelve treatments and five biological replicates per each treatment. The treatments were:

- T1: (control)
- T2: (1 mM Na₂SiO₃)
- T3: (2 mM Na₂SiO₃)
- T4: (3 mM Na₂SiO₃)
- T5 (75 mM NaCl)
- T6 (1 mM Na₂SiO₃ + 75 mM NaCl)
- T7 (2 mM Na₂SiO₃ + 75 mM NaCl)
- T8 (3 mM Na₂SiO₃ + 75 mM NaCl)
- T9 (150 mM NaCl)
- T10 (1 mM Na₂SiO₃ + 150 mM NaCl)
- T11 (2 mM Na₂SiO₃ + 150 mM NaCl)
- T12 (3 mM Na₂SiO₃ + 150 mM NaCl).

Seventy-five mM and 150 mM NaCl concentrations have been well documented in previous studies to represent moderate and high salinity stress conditions [20]. The 1, 2, and 3 mM sodium silicate (Na₂SiO₃) concentrations have been chosen for the graded application of Si through foliar sprays, from low to high rates [18].

### Evaluation of morphological and physiological characteristics

Plants at 45 DAS, i.e., 30 days after transplantation, were collected and assessed in terms of growth parameters. These included morphological parameters such as height, fresh weight of shoots, dry weight of shoots, fresh weight of roots, and dry weight of roots on a per-plant basis. Five biological replicates were utilized for each treatment in physiological and biochemical analyses.

### Determination of chlorophyll content

To extract chlorophyll (chl) pigments, 0.25 g of freshly cut leaf tissue was mixed in a Falcon tube with 80% acetone (10 mL). The leaf material was then left in the dark for three days until it became white. A spectrophotometer was used to measure the Chl solution at 470, 645, and 663 nm. The amounts of carotenoids, total pigment, Chl a, and Chl b were then measured using the methods described by [21].

### Determination of Relative Water Content (RWC)

RWC of leaves was calculated following the procedure given by Barrs and Weatherley [22]. Fully expanded and mature leaves were harvested from all treatments after completion of the experimental period. Fresh leaf samples were gently blotted with filter paper to wipe out any surface moisture, and fresh weight (FW) was noted on an analytical balance. The leaf samples were then submerged in distilled water and kept in the dark at 4 °C for 24 h to attain maximum turgidity. Leaf samples were taken out after attaining turgidity, surface moisture was wiped out gently with the help of filter paper, and turgid weight (TW) was measured. After that, the samples were dried in the oven at 70 °C for 48 h (until constant weight was attained), and dry weight (DW) was measured. The formula used to calculate relative water content is as follows:

$$\mathrm{RWC}\left(\%\right)=\left(\mathrm{FW}-\mathrm{DW}\right)/\left(\mathrm{TW}-\mathrm{DW}\right)\times100$$

### Determination of Membrane Stability Index (MSI)

MSI was assessed using leaf tissue materials. About 100 mg of leaves were chopped into short segments, gently cleaned with distilled H₂O, and introduced to test tubes containing distilled water. The samples were heated at 40 °C for 30 min in a water bath before measuring the initial electrical conductivity (C₁) with a conductivity meter. The collected values were used to determine the membrane’s stability. The tubes were first heated in a water bath at 100 °C for 10 min. The samples were then autoclaved at 120 °C for 20 min to completely damage the tissue. After cooling and reaching thermal equilibrium at 25 °C, the final conductivity value (C₂) was measured and utilized to calculate electrolyte leakage using the following formula [23].

$$\text{Membrane stability index}=\left(1-\mathrm{C1}/\mathrm{C2}\right)\times100$$

### Determination of Electrolyte Leakage (EL)

The technique outlined by Szalai et al. [24] was used to measure electrolyte leakage. Twenty leaf discs, each measuring one centimeter, were submerged in ten milliliters of distilled H_2_O and kept at ambient temperature for twenty hours while being gently shaken. First, the bathing solution’s conductivity (EC₁) was measured. The samples were then heated to 100 °C for 10 min to liberate all electrolytes, and the final conductivity (EC₃) was documented. The percentage of EL was determined using the following formula:

$$\mathrm{EL}\;\%=\left(\left(\text{EC2 - EC1}\right)/\mathrm{EC3}\right)\times100$$

### Determination of proline content

The estimation of free proline content was done as described by Bates et al. [25]. About 0.2 g of freshly excised leaves was ground with 4 mL of 3% (w/v) sulfosalicylic acid in a cooled mortar and pestle. The extract was filtered through Whatman No. 2 filter paper, and 2 mL of the filtrate was taken in a test tube. Afterwards, 2 mL of acid ninhydrin reagent and 2 mL of glacial acetic acid were added and kept in a boiling water bath (100 °C) for 1 h. After the heating process was completed, the tubes were quickly immersed in an ice bath for cooling. In each tube, 4 mL of toluene was added, and the contents were shaken thoroughly for 15–20 s to facilitate the extraction of the chromophore. The test tubes were kept undisturbed till complete phase separation occurred. The upper layer of toluene containing the chromophore was collected in another set of clean test tubes, and the absorbance was measured at 520 nm in a UV–Visible spectrophotometer using toluene as blank.

### Determination of Reactive Oxygen Species (ROS)

The concentration of hydrogen peroxide (H₂O₂) was established as per the protocol outlined by Velikova et al. [26]. About 0.2 g of fresh leaf tissue was ground in 4 mL of 0.1% (w/v) trichloroacetic acid (TCA) in a mortar that was pre-cooled. This homogenate was then centrifuged at 12,000 x g for 20 min at 4 °C, and the supernatant obtained was used for further analysis. To 0.8 mL of the supernatant, 0.8 mL of 10 mM potassium phosphate buffer (pH 7.0) and 0.4 mL of 1 M potassium iodide (KI) were added. The solution was mixed well and allowed to react in the dark at room temperature for some minutes. The absorption of the reaction mixture was measured at 390 nm using a UV-Vis spectrophotometer.

The formation of hydroxyl radical (•OH) was performed in accordance with the procedure described by Babbs et al. [27] through the utilization of the deoxyribose assay for radical formation. The reaction mixture (1 mL in total volume) consisted of 2-deoxy-D-ribose, FeCl₃, EDTA, KH₂PO₄–KOH buffer (20 mM, pH 7.4), H₂O₂, and ascorbic acid. After vigorous mixing of all the reagents, reaction mixture was incubated at 37 °C for 1 h. Thereafter, TBA and TCA were added and heated in a boiling water bath to generate the chromogen. After cooling to room temperature, the absorbance was measured at 532 nm with a UV–Vis spectrophotometer. Formation of hydroxyl radicals was assessed based on deoxyribose degradation.

### Determination of non-antioxidant enzymes

#### Ascorbic Acid (ASA) Analysis

The concentration of AsA was measured as described by Mukherjee and Choudhuri [28]. A sample of 500 mg of dried leaf tissue was extracted in 10 mL of 6% (w/v) trichloroacetic acid (TCA) and centrifuged at 12,000 × g for 15 min. The supernatant was then allowed to react with 2% (w/v) 2,4-dinitrophenylhydrazine (DNPH) containing thiourea and CuSO₄, followed by boiling in a water bath at 100 °C for 15 min. This was allowed to cool down to room temperature, and 80% (v/v) sulfuric acid (H₂SO₄) was slowly added into the mixture. The absorbance was read at 530 nm in a UV–Vis spectrophotometer. The concentration of ascorbic acid was determined using a standard curve made up of authentic L-ascorbic acid.

#### Reduced Glutathione (GSH) content

Determination of GSH content was carried out according to the procedure of Owens and Belcher [29]. Around 50 mg of the dried leaf tissue sample was powdered and extracted with 2 ml of 2% metaphosphoric acid solution (w/v). The extract was then centrifuged at 17,000 x g for 10 min at 4 °C, after which clear supernatant was isolated and used for determination of glutathione. The content of reduced glutathione was determined by the formation of a colored chromophore upon the reaction between reduced glutathione and DTNB (5,5′-dithiobis-(2-nitrobenzoic acid)), a yellow-colored compound that is also referred to as Ellman’s reagent. Absorbance of the reaction mixture was read at 412 nm using a UV-Visible spectrophotometer. Concentration of reduced glutathione was calculated from the standard curve prepared with reduced glutathione.

### Determination of antioxidant enzymes

The activities of the enzymes functioning as antioxidants were determined in the extracts of the leaves prepared as per the standard procedure. Fresh leaf samples (0.5 g) were homogenized in ice cold phosphate buffer solution (50 mM, pH 7.0) containing suitable enzyme stabilizers. The homogenate was then centrifuged at 12,000 x g for 20 min at 4 °C. The supernatant obtained from the centrifugation was used as a crude enzyme extract to determine the enzyme activities. Enzyme activities were determined as U mg^− 1^ protein.

Peroxidase (POX; EC 1.11.1.7) activity was measured following the procedure described by Vetter et al. [30] based on the oxidation of pyrogallol in the presence of hydrogen peroxide. The reaction mixture (3.0 mL total volume) contained 2.4 mL of 0.1 M phosphate buffer (pH 6.0), 0.3 mL of 5% (w/v) pyrogallol, 0.2 mL of 0.5% (v/v) hydrogen peroxide, and 0.1 mL of crude enzyme extract, with a blank prepared by substituting the enzyme extract with extraction buffer. The oxidation product purpurogallin was measured at 470 nm in a UV-VIS spectrophotometer, and one unit of POX activity was defined as the activity producing an absorbance increase of 0.01/min at 470 nm.

Superoxide dismutase (SOD; EC 1.15.1.1) activity was estimated using the standard nitroblue tetrazolium (NBT) photoreduction inhibition method [31, 32]. The reaction mixture (3.0 mL total volume) consisted of 50 mM potassium phosphate buffer (pH 7.8), 13 mM methionine, 75 µM NBT, 2 µM riboflavin, 0.1 mM EDTA, and an appropriate aliquot of enzyme extract. The reaction was initiated by illuminating the reaction mixtures under fluorescent lamps for 10–15 min, and the absorbance of the resulting formazan dye was recorded at 560 nm against a non-illuminated blank, where one unit of SOD activity was defined as the amount of enzyme causing a 50% inhibition of the photochemical reduction of NBT.

Catalase (CAT; EC 1.11.1.6) activity was quantified according to Abogadallah [33] in a 3.0 mL reaction mixture containing 2.8 mL of 50 mM potassium phosphate buffer (pH 7.0), 0.1 mL of 30 mM hydrogen peroxide, and 0.1 mL of enzyme extract against a buffer-peroxide blank. CAT was calculated as the reduction in absorbance due to hydrogen peroxide decomposition at 240 nm using the molar extinction coefficient ($\:39.4{\text{ mM}}^{-1}{\text{ cm}}^{-1}$).

Ascorbate peroxidase (APX; EC 1.11.1.11) activity was estimated as described by Nakano and Asada [34] using a 1.0 mL reaction mixture composed of 50 mM potassium phosphate buffer (pH 7.0), 0.5 mM ascorbic acid, 0.1 mM hydrogen peroxide, and 0.1 mL of enzyme extract (with a blank omitting hydrogen peroxide). The change in absorbance due to ascorbate oxidation was measured at 290 nm using an extinction coefficient of $\:2.8{\text{ mM}}^{-1}{\text{ cm}}^{-1}$, and APX activity was calculated in terms of $\:{\text{U mg}}^{-1}$ protein.

Glutathione reductase (GR; EC 1.6.4.2) activity was estimated as described by Jung et al. [35] using a 1.0 mL reaction mixture containing 100 mM potassium phosphate buffer (pH 7.8), 2 mM EDTA, 0.2 mM NADPH, 0.5 mM GSSG, and 0.1 mL of enzyme extract against a blank omitting GSSG. The decrease in absorbance due to the oxidation of NADPH to GSSG reduction was measured at 340 nm using an extinction coefficient of $\:6.2{\text{ mM}}^{-1}{\text{ cm}}^{-1}$, and GR activity was reported in $\:{\text{U mg}}^{-1}$ protein.

### Determination of elemental analysis

The amounts of the mineral elements were measured by following the procedure provided by Sen Tran et al. [36]. The leaves were first washed using distilled/deionized water in order to remove any Si that may have accumulated on the leaves. The cleaned leaves were dried and finely ground. About 100 mg of finely ground dried leaves were digested using concentrated nitric acid (13 M). Digestion was carried out at 185 °C for 25 min. After digestion, the extract was cooled down, diluted to the required volume with ultrapure water, and further used for determination of the amounts of elements.

The amounts of phosphorus (P), potassium (K), sodium (Na), and silicon (Si) were measured by inductively oupled plasma-optical emission spectrometry (ICP-OES) and inductively with a glass nebulizer with a flow rate of 0.4 mL min⁻¹. Calibration curves were obtained using external standard solutions in the concentration range from 1 to 600 µg L⁻¹. The standards and samples were prepared in 0.23 M nitric acid solution to reduce the effect of the matrix. The amounts of P, K, Na, and Si were determined from the calibration curve and presented as mg g⁻¹ DW. The total N content was measured using the micro-Kjeldahl method after [37]. About 100 mg of dried and powdered leaf samples were digested using concentrated sulfuric acid along with certain catalysts. Ammonia gas generated during digestion was collected and measured using a micro-Kjeldahl apparatus (Ningbo Medical Instruments Co., Ningbo, China). N content was then calculated from titration results.

### Statistical analysis

Data were examined using SPSS (Social Science Version 27.00) under a completely randomized design. Two-way was used, followed by Tukey’s post hoc test. Homogeneity of variance was assessed using Levene’s test. Statistical significance was considered at *p* ≤ 0.05, corresponding to a 95% confidence level. Pearson’s correlation analysis was performed among all measured variables using the treatment means (*n* = 12). Pearson’s correlation coefficients (r) and corresponding two-tailed p-values were calculated, with statistical significance considered at *p* < 0.05.

For multivariate analysis, all variables were standardized prior to analysis using Z-score transformation, whereby each observation was centered at a mean of zero and scaled to a standard deviation of one. Standardization was applied to eliminate the influence of differences in measurement units and numerical magnitude among variables. Principal component analysis (PCA) was then conducted on the standardized dataset. The percentage of variance explained by each principal component was calculated, and correlation loadings between the original standardized variables and the principal component scores were used to identify the variables contributing most strongly to PC1 and PC2. Hierarchical cluster analysis (HCA) was performed using the same standardized dataset. Euclidean distance was used as the dissimilarity measure, and Ward’s minimum-variance method was applied for agglomerative linkage. The major cluster structure was determined from the dendrogram based on the pronounced increase in linkage distance preceding the final cluster fusion.

## Results

### Effect of silicon on growth parameters under salt stress

**Fig. 1:**
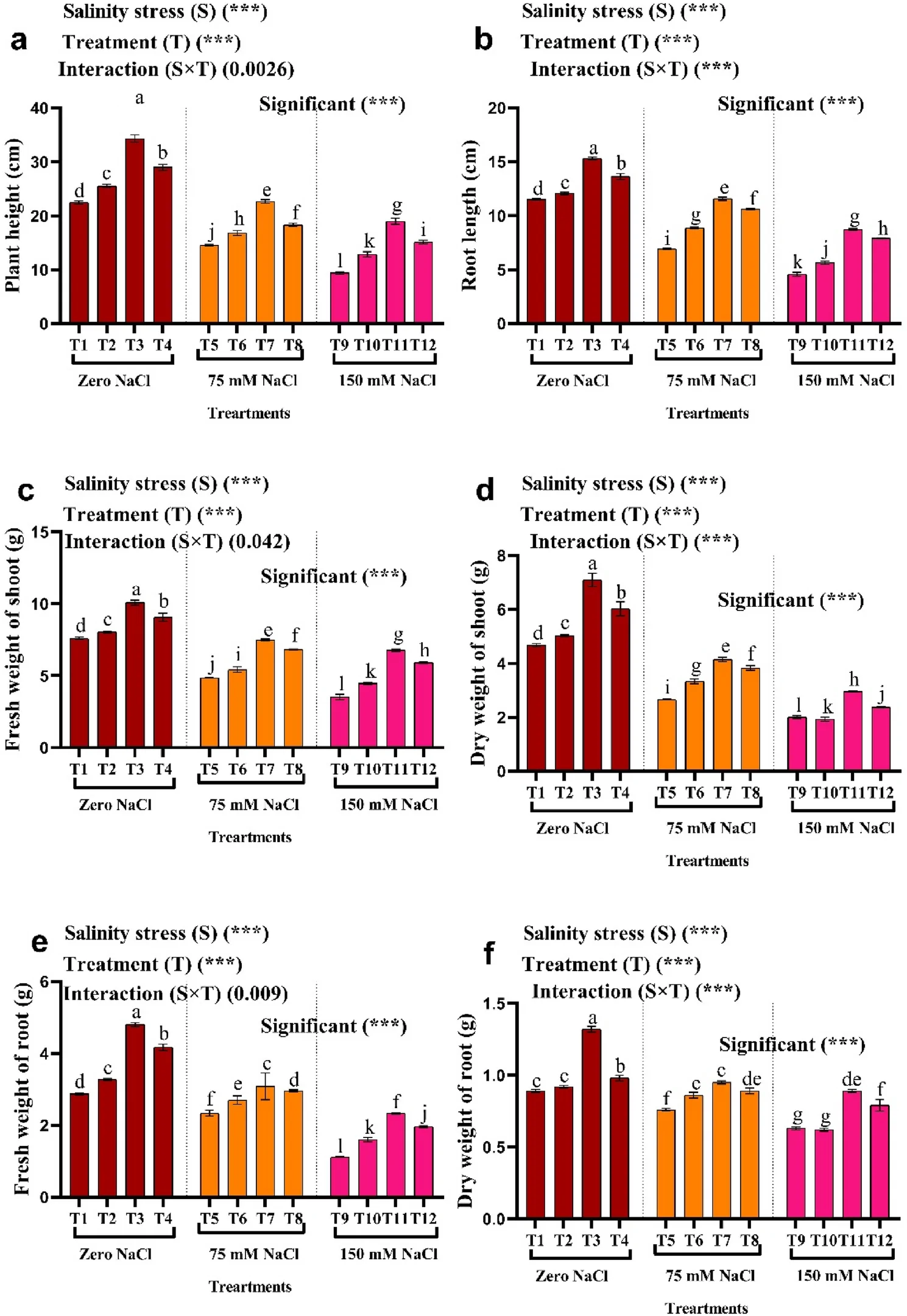
Effects of foliar application of silicon on (**a**) shoot length, (**b**) root length, (**c**) shoot fresh weight, (**d**) shoot dry weight, (**e**) root fresh weight, and (**f**) root dry weight of tomato plants exposed to NaCl stress. Values are means ± standard error (SE) of replicates. The superscript letters in bars represent significant differences between treatments based on two-way ANOVA followed by Tukey’s HSD test at *p* ≤ 0.05. The symbols represent statistical significance at the following probability levels: *** *p* < 0.001. T1: (control), T2: (1 mM Na₂SiO₃), T3: (2 mM Na₂SiO₃), T4: (3 mM Na₂SiO₃), T5: (75 mM NaCl), T6: (1 mM Na₂SiO₃ + 75 mM NaCl), T7: (2 mM Na₂SiO₃ + 75 mM NaCl), T8: (3 mM Na₂SiO₃ + 75 mM NaCl), T9: (150 mM NaCl), T10: (1 mM Na₂SiO₃ + 150 mM NaCl), T11: (2 mM Na₂SiO₃ + 150 mM NaCl), T12: (3 mM Na₂SiO₃ + 150 mM NaCl)

The growth parameters of tomato plants were significantly influenced by NaCl salt concentration and sodium silicate treatment (Fig. 1a-f). In the absence of salinity stress, the control group plants had poorer growth traits when compared to the plants treated with sodium silicate. The highest value of growth traits was obtained by 2 mM Na₂SiO₃, where the height of the plant was measured at 34.0 cm, root length at 15.2 cm, shoot fresh weight at 10.0 g, shoot dry weight at 7.1 g, root fresh weight at 4.8 g, and root dry weight at 1.3 g. At a high NaCl concentration of 75 mM, growth parameters declined significantly when compared to the control. The height of the plant, root length, shoot fresh and dry weights, and root fresh and dry weights decreased to 14.5 cm, 6.8 cm, 4.8 g, 2.6 g, 2.3 g, and 0.8 g, respectively. The 2 mM Na₂SiO₃ solution increased these growth parameters to 22.5 cm, 11.5 cm, 7.5 g, 4.1 g, 3.1 g, and 1.0 g, respectively.

Plant growth was significantly inhibited by 150 mM NaCl, with plant height, root length, shoot fresh and dry weight, and root fresh and dry weight values measured as 9.2 cm, 4.5 cm, 3.5 g, 2.0 g, 1.1 g, and 0.6 g, respectively. Addition of 1 mM Na₂SiO₃ resulted in an improvement in most growth parameters compared to that of 150 mM NaCl. Growth inhibition due to salinity was significantly minimized when sodium silicate concentration reached 2 mM, resulting in 20.0 cm of plant height, 8.6 cm of root length, 7.0 g of shoot fresh weight, 4.2 g of shoot dry weight, 2.4 g of root fresh weight, and 0.9 g of root dry weight, respectively. Although 3 mM of Na₂SiO₃ also significantly increased growth compared to 150 mM NaCl, it was less effective than 2 mM of Na₂SiO₃. Overall, increasing NaCl stress gradually inhibited tomato growth, with the highest degree of reduction at 150 mM NaCl stress level. Na₂SiO₃ effectively mitigated these negative impacts on tomato plants, and 2 mM of Na₂SiO₃ had the most growth-promoting effect.

### Effect of silicon on photosynthetic characteristics unser salt stress

**Fig. 2:**
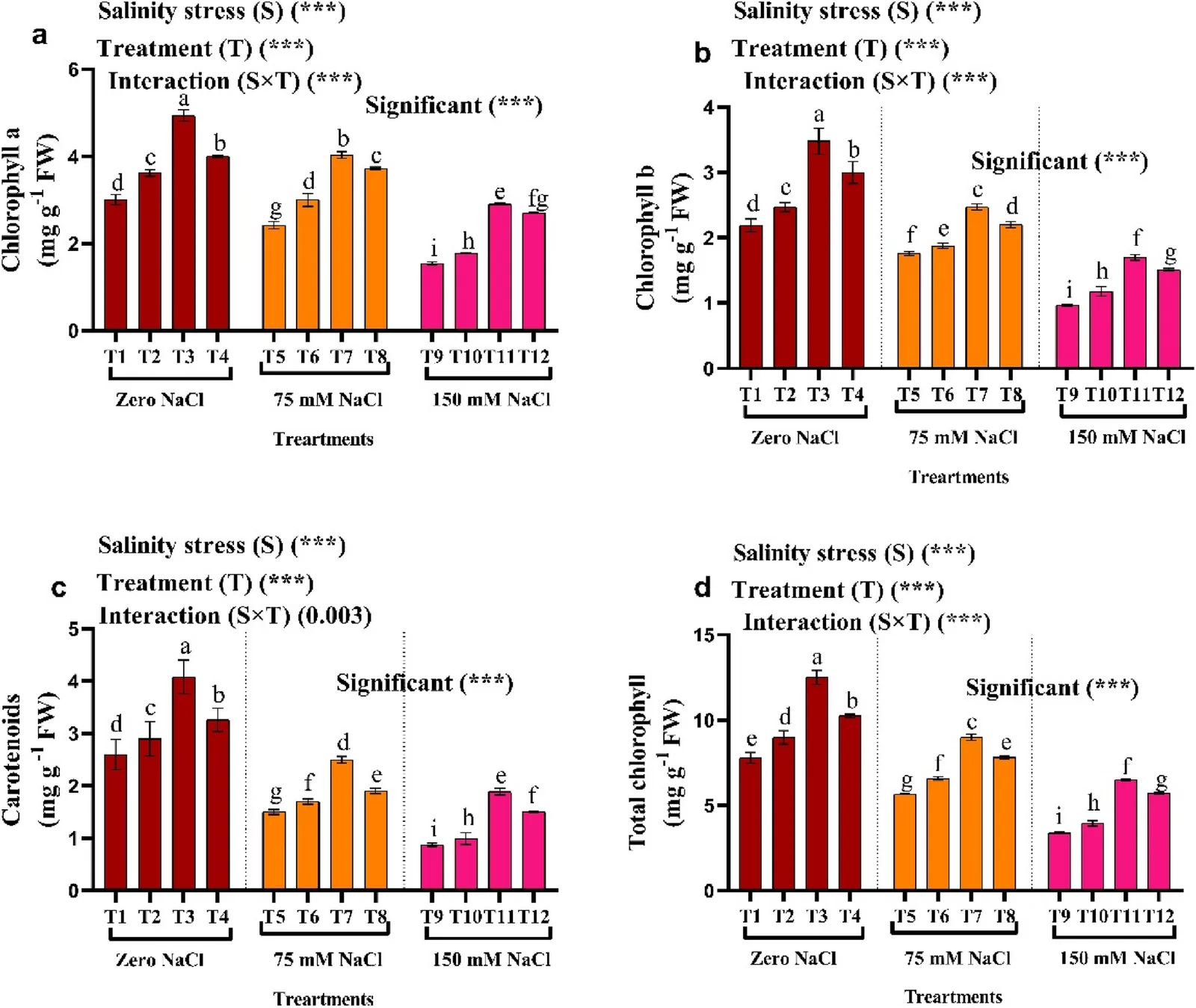
Effects of foliar application of silicon on (**a**) chlorophyll a, (**b**) chlorophyll b, (**c**) carotenoids, and (**d**) total pigments in leaves of tomato plants exposed to NaCl stress. Values are means ± Standard Error (SE) of replicates. The superscript letters in bars represent significant differences between treatments based on two-way ANOVA followed by Tukey’s HSD test at *p* ≤ 0.05. The symbols represent statistical significance at the following probability levels: *** *p* < 0.001. T1: (control), T2: (1 mM Na₂SiO₃), T3: (2 mM Na₂SiO₃), T4: (3 mM Na₂SiO₃), T5: (75 mM NaCl), T6: (1 mM Na₂SiO₃ + 75 mM NaCl), T7: (2 mM Na₂SiO₃ + 75 mM NaCl), T8: (3 mM Na₂SiO₃ + 75 mM NaCl), T9: (150 mM NaCl), T10: (1 mM Na₂SiO₃ + 150 mM NaCl), T11: (2 mM Na₂SiO₃ + 150 mM NaCl), T12: (3 mM Na₂SiO₃ + 150 mM NaCl)

The effect of salinity on photosynthetic pigment content was highly significant, as shown in Fig. 2a–d. At non-saline conditions, the highest amounts of chlorophyll a (5.0 mg g⁻¹ FW), chlorophyll b (3.5 mg g⁻¹ FW), carotenoids (4.1 mg g⁻¹ FW), and total chlorophyll (12.5 mg g⁻¹ FW) were found in 2 mM Na₂SiO₃. Under 75 mM NaCl, the amounts of chlorophyll a, chlorophyll b, carotenoids, and total chlorophyll decreased to 2.4, 1.8, 1.5, and 5.7 mg g⁻¹ FW, respectively. Treatment with 2 mM Na₂SiO₃ in the presence of 75 mM NaCl increased these parameters to 4.1, 2.5, 2.5, and 9.0 mg g⁻¹ FW, respectively. When exposed to 150 mM NaCl without 2 mM Na₂SiO₃, pigment levels fell even lower to 1.5, 1.0, 0.9, and 3.4 mg g⁻¹ FW of chlorophyll a, chlorophyll b, carotenoids, and total chlorophyll, respectively. In contrast, when 2 mM Na₂SiO₃ was added, these amounts were increased to 2.9, 1.7, 1.9, and 6.6 mg g⁻¹ FW, respectively.

In general, 2 mM Na₂SiO₃ was the most effective treatment, especially under saline conditions, since it could significantly increase chlorophyll pigments and carotenoid levels. Thus, it seems likely that the addition of silicon is beneficial for the protection of the photosynthetic system and salt tolerance in tomato.

### Effect of silicon on electrolyte leakage, lipid peroxidation, content, and ROS production under salt stress

**Fig. 3:**
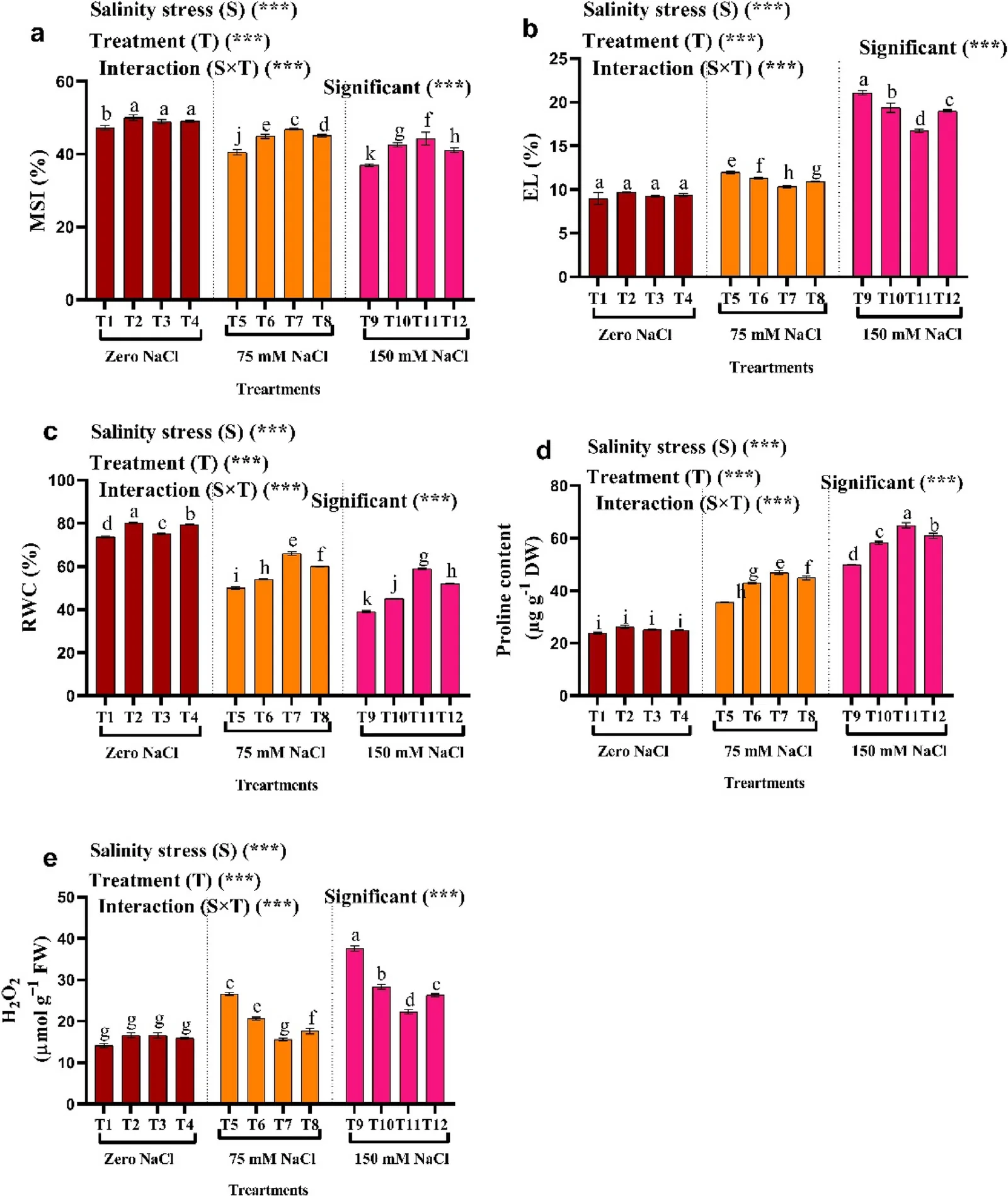
Effects of foliar application of silicon on (**a**) MSI, (**b**) EL, (**c**) RWC, (**d**) proline, and (**e**) H₂O₂ in leaves of tomato plants exposed to NaCl stress. Values are means ± Standard Error (SE) of replicates. The superscript letters in bars represent significant differences between treatments based on two-way ANOVA followed by Tukey’s HSD test at *p* ≤ 0.05. The symbols represent statistical significance at the following probability levels: *** *p* < 0.001. T1: (control), T2: (1 mM Na₂SiO₃), T3: (2 mM Na₂SiO₃), T4: (3 mM Na₂SiO₃), T5: (75 mM NaCl), T6: (1 mM Na₂SiO₃ + 75 mM NaCl), T7: (2 mM Na₂SiO₃ + 75 mM NaCl), T8: (3 mM Na₂SiO₃ + 75 mM NaCl), T9: (150 mM NaCl), T10: (1 mM Na₂SiO₃ + 150 mM NaCl), T11: (2 mM Na₂SiO₃ + 150 mM NaCl), T12: (3 mM Na₂SiO₃ + 150 mM NaCl)

The presence of NaCl salinity had a negative impact on membrane stability and water content in plants, which caused an increase in proline and H₂O₂ levels (Figs. 3a–e). The presence of 75 mM NaCl resulted in decreases in MSI and RWC to 41.0% and 50.0%, respectively, and increases in EL, proline, and H₂O₂ to 12.0%, 35.5 µg g⁻¹ DW, and 26.5 µmol g⁻¹ FW, respectively. In the case of 150 mM NaCl, the effect of NaCl on plants was more pronounced as compared to 75 mM NaCl, and MSI and RWC decreased to 37.0% and 39.5%, while EL, proline, and H₂O₂ increased to 21.0%, 50.0 µg g⁻¹ DW, and 37.5 µmol g⁻¹ FW, respectively.

Application of 2 mM Na₂SiO₃ proved to be the best treatment at both salinity regimes. At 75 mM NaCl, it raised MSI and RWC to 47.5% and 68.0%, respectively, and decreased EL and H₂O₂ levels to 10.5% and 15.8 µmol g⁻¹ FW and increased proline content up to 47.0 µg g⁻¹ DW. At 150 mM NaCl, 2 mM Na₂SiO₃ also improved MSI and RWC to 44.2% and 59.0%, respectively, decreased EL and H₂O₂ levels to 16.8% and 22.2 µmol g⁻¹ FW, and raised proline content to 64.5 µg g⁻¹ DW. In general, 2 mM Na₂SiO₃ was able to minimize the harmful effects of salinity on the plasma membrane, minimize water loss, enhance proline content, and decrease H₂O₂ production.

### Effect of silicon on non-enzymatic antioxidants under salt stress

**Fig. 4:**
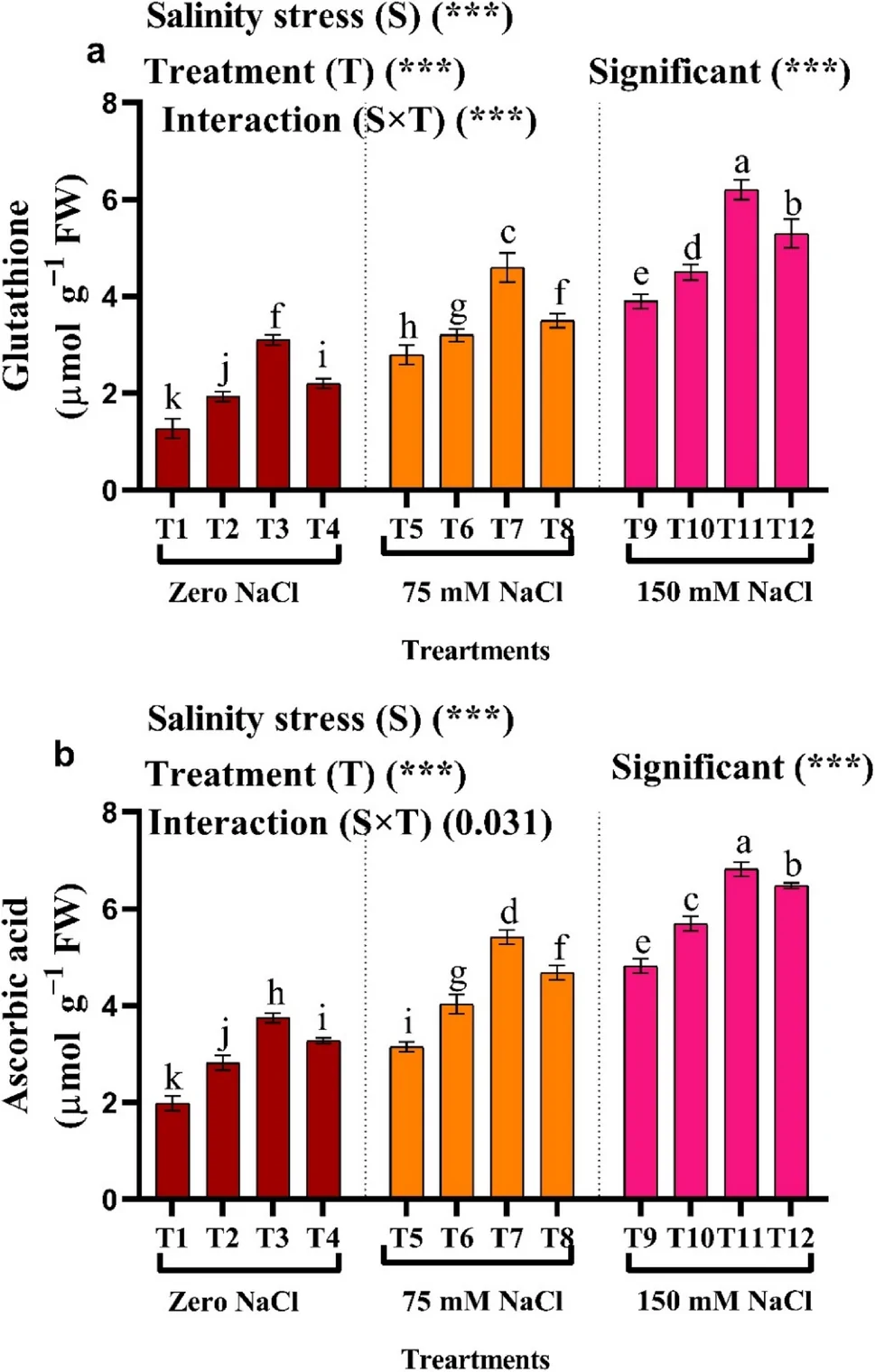
Effects of foliar application of silicon on (**a**) glutathione and (**b**) ascorbic acid in leaves of tomato plants exposed to NaCl stress. Values are means ± Standard Error (SE) of replicates. The superscript letters in bars represent significant differences between treatments based on two-way ANOVA followed by Tukey’s HSD test at *p* ≤ 0.05. The symbols represent statistical significance at the following probability levels: *** *p* < 0.001. T1: (control), T2: (1 mM Na₂SiO₃), T3: (2 mM Na₂SiO₃), T4: (3 mM Na₂SiO₃), T5: (75 mM NaCl), T6: (1 mM Na₂SiO₃ + 75 mM NaCl), T7: (2 mM Na₂SiO₃ + 75 mM NaCl), T8: (3 mM Na₂SiO₃ + 75 mM NaCl), T9: (150 mM NaCl), T10: (1 mM Na₂SiO₃ + 150 mM NaCl), T11: (2 mM Na₂SiO₃ + 150 mM NaCl), T12: (3 mM Na₂SiO₃ + 150 mM NaCl)

The increase in NaCl salinity caused an increase in the content of glutathione and ascorbic acid in comparison with the control, which were higher at 150 mM NaCl than at 75 mM (Fig. 4a, b). At 75 mM NaCl, glutathione and ascorbic acid accumulated to 2.8 and 3.1 µmol g⁻¹ FW, while under 150 mM NaCl these levels were 3.9 and 4.8 µmol g⁻¹ FW, respectively. However, the application of 2 mM Na₂SiO₃ proved to be the most efficient treatment in increasing antioxidant activity in both cases. In case of 75 mM NaCl salinity, 2 mM Na₂SiO₃ increased the level of glutathione and ascorbic acid to 4.6 and 5.4 µmol g⁻¹ FW, while under 150 mM NaCl, these values increased to 6.2 and 6.8 µmol g⁻¹ FW, respectively. Thus, 2 mM Na₂SiO₃ enhanced the antioxidant capacity of tomato plants under both moderate and severe salinity. The results confirm that silicon improves the ability of tomato plants to detoxify reactive oxygen species.

### Effect of silicon on antioxidant enzymes under salt stress

**Fig. 5:**
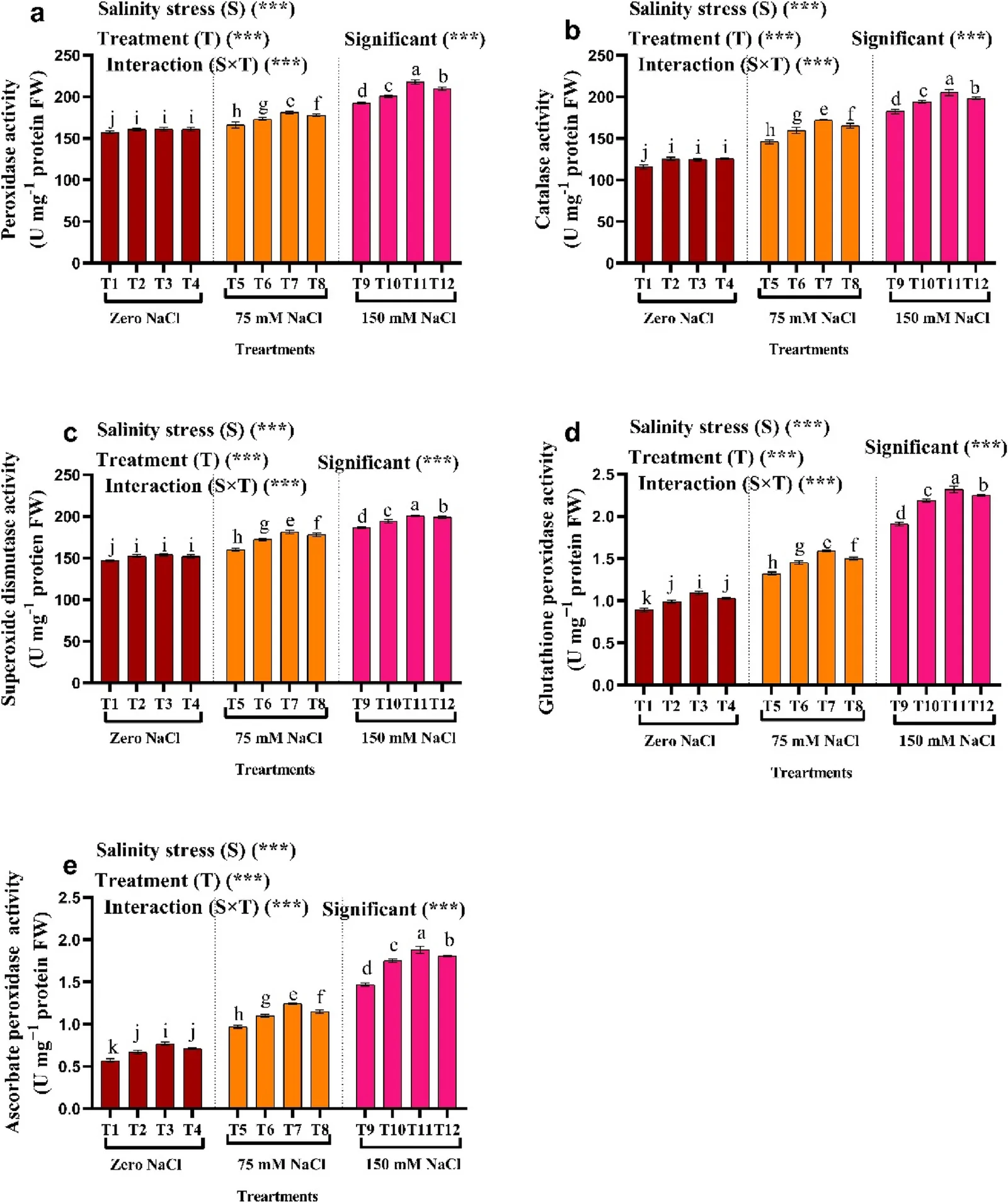
Effects of foliar application of silicon on (**a**) peroxidase, (**b**) catalase, (**c**) superoxide dismutase, (**d**) glutathione reductase, and (**e**) ascorbate peroxidase in leaves of tomato plants exposed to NaCl stress. Values are means ± standard error (SE) of replicates. The superscript letters in bars represent significant differences between treatments based on two-way ANOVA followed by Tukey’s HSD test at *p* ≤ 0.05. The symbols represent statistical significance at the following probability levels: *** *p* < 0.001. T1: (control), T2: (1 mM Na₂SiO₃), T3: (2 mM Na₂SiO₃), T4: (3 mM Na₂SiO₃), T5: (75 mM NaCl), T6: (1 mM Na₂SiO₃ + 75 mM NaCl), T7: (2 mM Na₂SiO₃ + 75 mM NaCl), T8: (3 mM Na₂SiO₃ + 75 mM NaCl), T9: (150 mM NaCl), T10: (1 mM Na₂SiO₃ + 150 mM NaCl), T11: (2 mM Na₂SiO₃ + 150 mM NaCl), T12: (3 mM Na₂SiO₃ + 150 mM NaCl)

The activities of all studied antioxidant enzymes were increased by NaCl salinity, and higher activities were recorded under 150 mM NaCl compared to those under 75 mM NaCl (Figs. 5a-e). Under 75 mM NaCl alone, the activities of peroxidase, catalase, superoxide dismutase, glutathione peroxidase, and ascorbate peroxidase enzymes were 166.0, 145.0, 160.0, 1.32, and 0.97 U mg⁻¹ protein FW, respectively. Under 150 mM NaCl alone, the activities of these enzymes were increased to 196.0, 182.0, 186.0, 1.91, and 1.46 U mg⁻¹ protein FW, respectively. The application of 2 mM Na₂SiO₃ proved to be the most effective treatment in both salinities. Under 75 mM NaCl, the application of 2 mM Na₂SiO₃ increased the activities of peroxidase, catalase, superoxide dismutase, glutathione peroxidase, and ascorbate peroxidase enzymes to 181.5, 173.0, 185.0, 1.60, and 1.24 U mg⁻¹ protein FW, respectively.

The application of 2 mM Na₂SiO₃ to plants grown in NaCl concentrations up to 150 mM led to the maximal activity of the antioxidant enzymes being reached: 218.0 U mg⁻¹ protein FW of peroxidase, 205.0 U mg⁻¹ protein FW of catalase, 201.0 U mg⁻¹ protein FW of superoxide dismutase, 2.30 U mg⁻¹ protein FW of glutathione peroxidase, and 1.88 U mg⁻¹ protein FW of ascorbate peroxidase. In summary, 2 mM Na₂SiO₃ proved to be the most efficient method of increasing the activities of antioxidant enzymes at both salinity concentrations; maximal values were achieved when 150 mM NaCl and 2 mM Na₂SiO₃ were used.

### Effect of silicon on mineral ion contents under salt stress

**Fig. 6:**
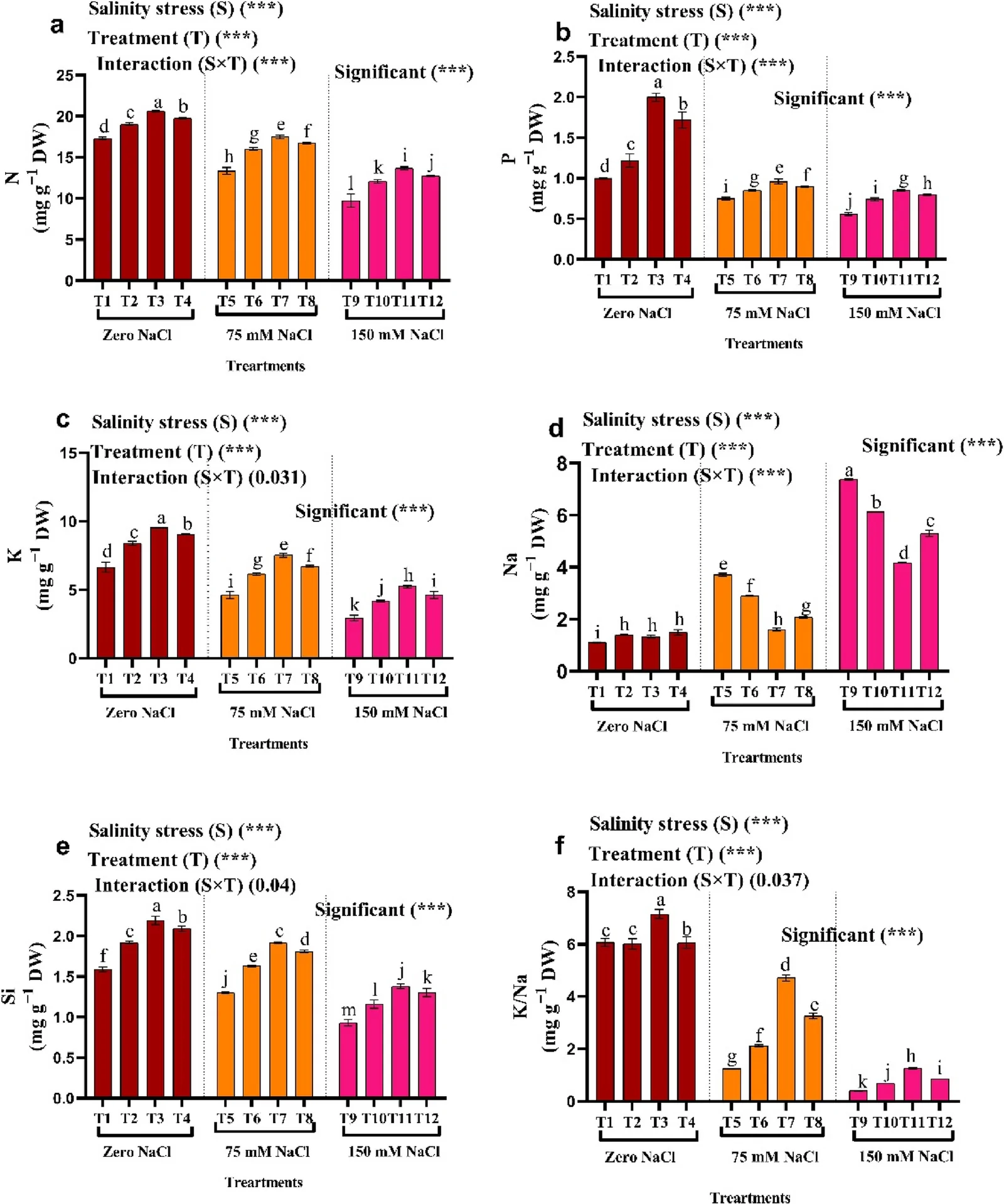
Effects of foliar application of silicon on (**a**) N, (**b**) P, (**c**) K, (**d**) Na, and (**e**) Si in leaves of tomato plants exposed to NaCl stress. Values are means ± standard error (SE) of replicates. The superscript letters in bars represent significant differences between treatments based on two-way ANOVA followed by Tukey’s HSD test at *p* ≤ 0.05. The symbols represent statistical significance at the following probability levels: *** *p* < 0.001. T1: (control), T2: (1 mM Na₂SiO₃), T3: (2 mM Na₂SiO₃), T4: (3 mM Na₂SiO₃), T5: (75 mM NaCl), T6: (1 mM Na₂SiO₃ + 75 mM NaCl), T7: (2 mM Na₂SiO₃ + 75 mM NaCl), T8: (3 mM Na₂SiO₃ + 75 mM NaCl), T9: (150 mM NaCl), T10: (1 mM Na₂SiO₃ + 150 mM NaCl), T11: (2 mM Na₂SiO₃ + 150 mM NaCl), T12: (3 mM Na₂SiO₃ + 150 mM NaCl)

The NaCl salinity had a significant effect on the uptake of nutrients and ions by tomato plants since there was reduced N, P, and K content coupled with an increase in Na+ content and a lower K^+^/Na^+^ ratio (Fig. 6a-f). At 75 mM NaCl concentration, there was a decrease in N, P, and K to 13.5, 0.8, and 4.8 mg g⁻¹ DW, respectively, while there was an increase in Na to 3.7 mg g⁻¹ DW, with a K^+^/Na^+^ ratio of 1.3. In the case of 150 mM NaCl, there was a higher reduction in N, P, and K to 9.7, 0.55, and 3.0 mg g⁻¹ DW, respectively, while Na⁺ and K⁺/Na⁺ were 7.4 mg g⁻¹ DW and 0.4 mg g⁻¹ DW, respectively. The application of 2 mM Na₂SiO₃ was the most effective method for the improvement of the mineral nutrition and ion homeostasis under both salt concentrations. With the application of 75 mM NaCl, 2 mM Na₂SiO₃ increased the concentration of N, P, and K to 17.5, 0.95, and 8.0 mg g⁻¹ DW, respectively, and decreased Na accumulation to 1.6 mg g⁻¹ DW, while the Si concentration and the K/Na ratio were increased to 1.9 mg g⁻¹ DW and 4.8, respectively. On the application of 150 mM NaCl, 2 mM Na₂SiO₃ increased the N, P, and K to 13.5, 0.90, and 5.4 mg g⁻¹ DW, respectively, decreased Na accumulation to 4.2 mg g⁻¹ DW, and increased the Si concentration and K^+^/Na^+^ ratio to 1.4 mg g⁻¹ DW and 1.5 mg g⁻¹ DW, respectively. In summary, 2 mM Na₂SiO₃ was the most effective treatment in alleviating the adverse effect of salinity on mineral nutrition and ion homeostasis.

### Correlation analysis

**Fig. 7:**
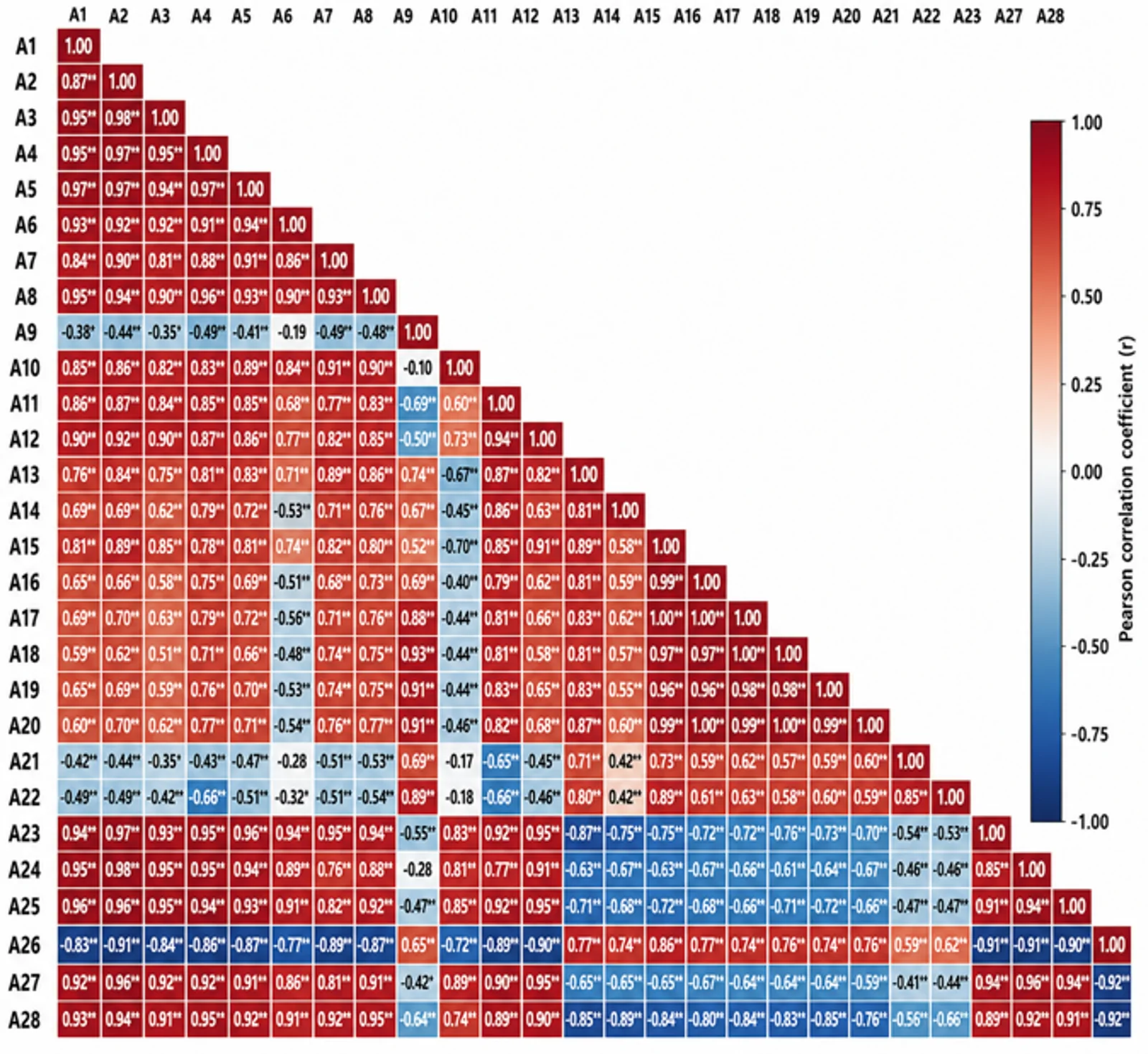
Heatmaps illustrate how quantitative statistics are related to mean values of various parameters in this study. Different parameters are represented on the color scale based on their normalized mean value. A1: Plant height, A2: Root length, A3: FW shoot, A4: DW shoot, A5: FW root, A6: DW root, A7: chl a, A8: chl b, A9: Carotenoids, A10: Total pigment, A11: RWC, A12: MSI, A13: EL, A14: Proline, A15: H₂O₂, A16: SOD, A17: CAT, A18: POX, A19: GR, A20: APX, A21: ASA, A22: GSH, A23: N, A24: P, A25: K, A26: Na, A27: Si, A28: K/Na

The pearson correlation matrix revealed clear interrelationships among growth, physiological, biochemical, and elemental traits (Fig. 7). Growth-related parameters, including plant height, root length, shoot and root biomass, photosynthetic pigments, relative water content (RWC), and membrane stability index (MSI), were strongly and positively correlated with each other, indicating their close association with overall plant vigor. For example, the K^+^/Na^+^ ratio was positively correlated with shoot dry weight (*r* = 0.955, *p* < 0.001), root length (*r* = 0.944, *p* < 0.001), N content (*r* = 0.942, *p* < 0.001), K content (*r* = 0.933, *p* < 0.001), and plant height (*r* = 0.926, *p* < 0.001). Likewise, Si accumulation showed strong positive correlations with K (*r* = 0.987, *p* < 0.001), N (*r* = 0.980, *p* < 0.001), root length (*r* = 0.964, *p* < 0.001), and root fresh weight (*r* = 0.959, *p* < 0.001).

In contrast, stress-associated traits were negatively related to growth and nutrient acquisition. Na accumulation was strongly and positively correlated with electrolyte leakage (*r* = 0.973, *p* < 0.001) and H₂O₂ (*r* = 0.963, *p* < 0.001), whereas it was negatively correlated with N (*r* = − 0.942, *p* < 0.001), root length (*r* = − 0.905, *p* < 0.001), Si accumulation (*r* = − 0.905, *p* < 0.001), K content (*r* = − 0.898, *p* < 0.001), and the K^+^/Na^+^ ratio (*r* = − 0.886, *p* < 0.001). These relationships demonstrate that salinity stress was associated with higher Na accumulation, oxidative damage, and membrane injury, whereas improved Si status and K^+^/Na^+^ balance were associated with better growth performance and nutrient homeostasis.

### Principal component analysis

**Fig. 8:**
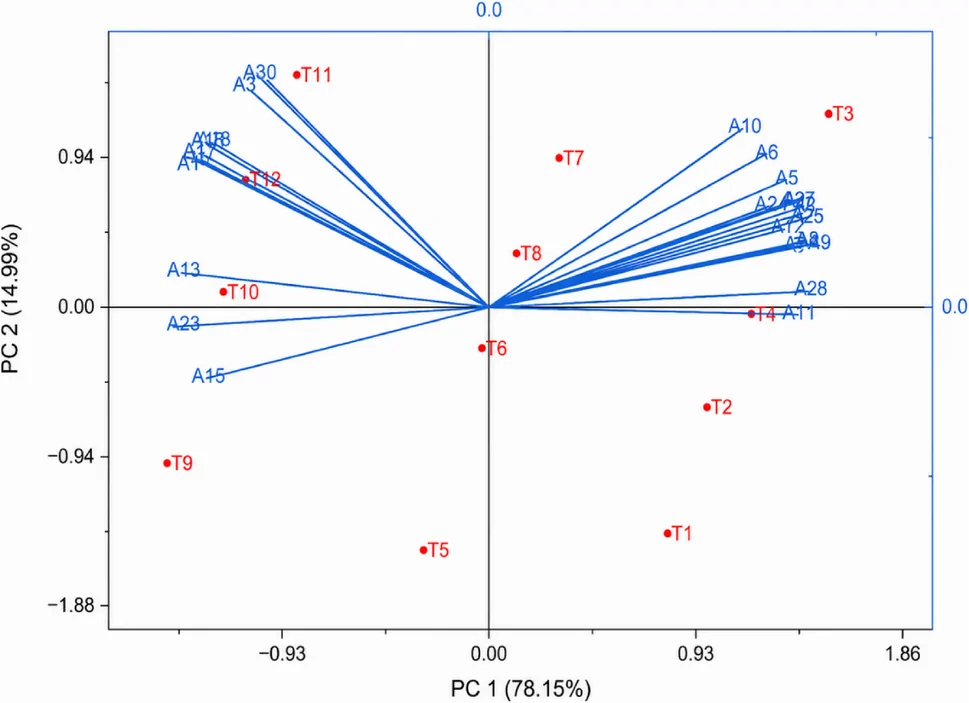
Principal Component Analysis (PCA) loading plot for treatment application under control and treatments. T1: (control), T2: (1 mM Na₂SiO₃), T3: (2 mM Na₂SiO₃), T4: (3 mM Na₂SiO₃), T5: (75 mM NaCl), T6: (1 mM Na₂SiO₃ + 75 mM NaCl), T7: (2 mM Na₂SiO₃ + 75 mM NaCl), T8: (3 mM Na₂SiO₃ + 75 mM NaCl), T9: (150 mM NaCl), T10: (1 mM Na₂SiO₃ + 150 mM NaCl), T11: (2 mM Na₂SiO₃ + 150 mM NaCl), T12: (3 mM Na₂SiO₃ + 150 mM NaCl)

Principal component analysis (PCA) was performed using the standardized dataset to evaluate the multivariate relationships among the twelve treatments (T1–T12) and the measured parameters (A1–A28). The first principal component (PC1) explained 78.15% of the total variance, whereas the second principal component (PC2) explained 14.99%, together accounting for 93.14% of the total dataset variation (Fig. 8).

PC1 was mainly associated with positive loadings for N (0.972), K^+^/Na^+^ ratio (0.965), shoot dry weight (0.959), Chl b (0.953), K (0.952), root length (0.944), root fresh weight (0.940), RWC (0.928), Si (0.926), and plant height (0.921). In contrast, Na (− 0.933), electrolyte leakage (− 0.930), APX (− 0.889), CAT (− 0.885), GR (− 0.879), proline (− 0.876), SOD (− 0.858), and H₂O₂ (− 0.856) loaded negatively on PC1. PC2 was mainly influenced by AsA (0.704), carotenoids (0.695), GSH (0.667), total pigments (0.541), POX (0.501), and SOD (0.496).

At the treatment level, T3 showed the highest positive PC1 score, followed by T4 and T2, indicating their close association with favorable growth, pigment accumulation, nutrient status, and ion homeostasis. By contrast, T9 exhibited the most negative PC1 score, followed by T10, T12, and T11, reflecting their association with Na accumulation, oxidative stress, and membrane damage. Thus, the PCA clearly separated the non-stressed and Si-treated treatments from the severely salt-stressed treatments and highlighted the beneficial effect of sodium metasilicate in mitigating salinity-induced damage.

### Hierarchical cluster analysis

**Fig. 9:**
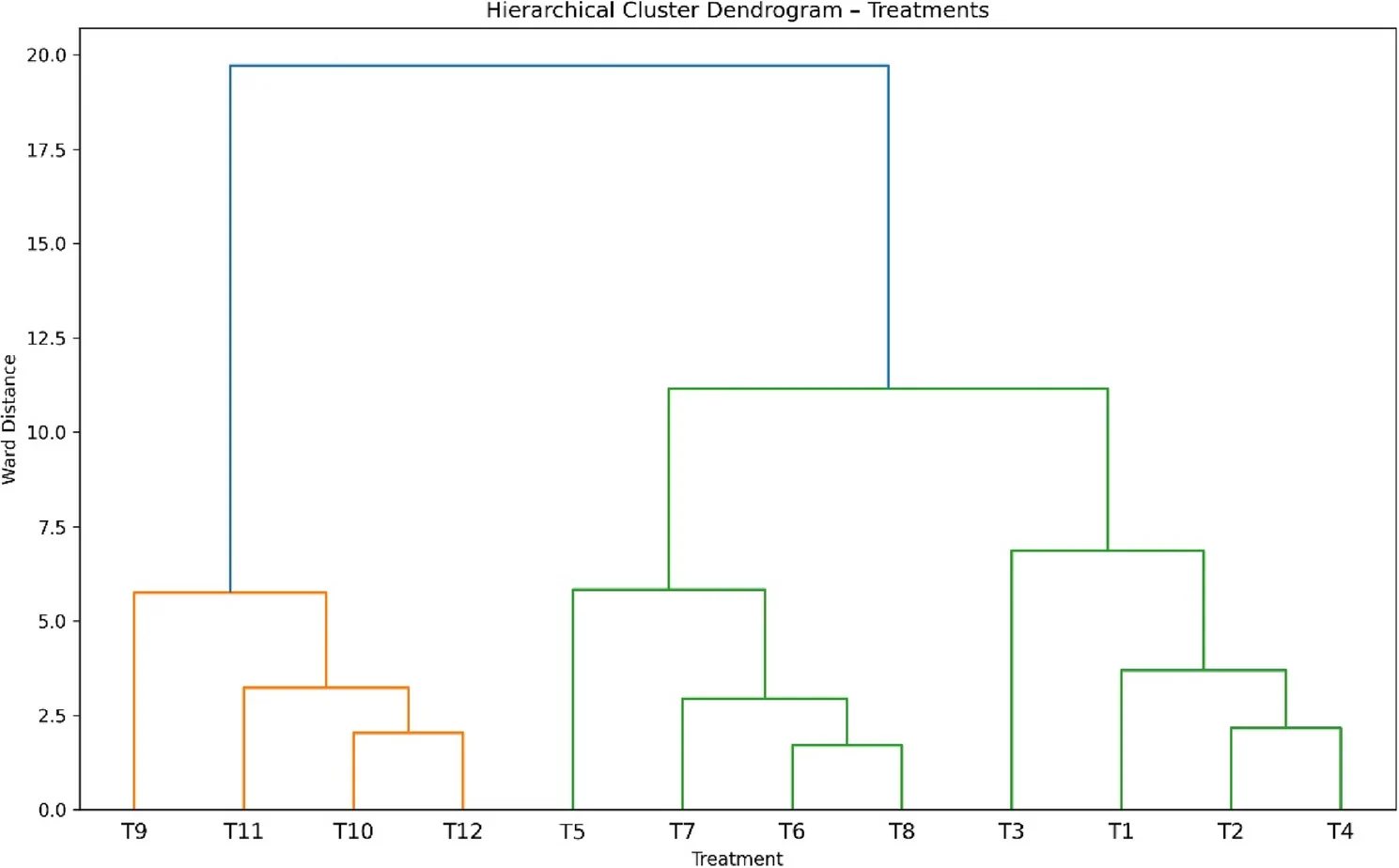
Hierarchical Clustering (HCA) for treatment application under control and treatments. T1: (control), T2: (1 mM Na₂SiO₃), T3: (2 mM Na₂SiO₃), T4: (3 mM Na₂SiO₃), T5: (75 mM NaCl), T6: (1 mM Na₂SiO₃ + 75 mM NaCl), T7: (2 mM Na₂SiO₃ + 75 mM NaCl), T8: (3 mM Na₂SiO₃ + 75 mM NaCl), T9: (150 mM NaCl), T10: (1 mM Na₂SiO₃ + 150 mM NaCl), T11: (2 mM Na₂SiO₃ + 150 mM NaCl), T12: (3 mM Na₂SiO₃ + 150 mM NaCl)

Hierarchical cluster analysis was conducted using standardized data, Euclidean distance, and Ward’s minimum-variance linkage method (Fig. 9). The dendrogram revealed a clear separation of the twelve treatments into two major clusters, with a marked increase in linkage distance before the final fusion, supporting the robustness of this grouping.

The first major cluster comprised T9, T11, T10, and T12, representing the treatments exposed to severe salinity (150 mM NaCl). Within this cluster, T9 was clearly separated from the sodium metasilicate-treated treatments (T10, T11, and T12), indicating that sodium metasilicate modified the response pattern under high salinity stress. The second major cluster included the remaining treatments and was further subdivided into two subclusters. The first subcluster contained T5, T7, T6, and T8, corresponding to the moderate salinity treatments (75 mM NaCl), whereas the second subcluster comprised T3, T1, T2, and T4, which represented the non-saline treatments. This clustering pattern was consistent with the PCA results and confirmed that salinity level was the main factor governing treatment segregation, while sodium metasilicate supplementation shifted the stressed treatments toward a less severe response pattern.

## Discussion

Salinity stress has been considered one of the major abiotic stresses that limit the growth and productivity of plants. Accumulation of salts through osmotic stress, ionic toxicity due to the accumulation of Na⁺ and Cl⁻; disruption of nutrients, inhibition of photosynthesis, and oxidative damages by the production of ROS are some mechanisms leading to the reduction in plant growth and yields [38]. In the current study, the increase in the concentration of NaCl decreased the plant height and biomass production. Such inhibitory effects of the salinity treatment on the growth of tomato were observed due to inhibited cell division and elongation, low turgor pressure, limited nutrient uptake, and metabolic disturbances caused by salinity. Reduction in tomato growth under salt stress conditions has also been reported extensively [39, 40].

On the other hand, exogenous addition of Na₂SiO₃ greatly increased all growth parameters irrespective of the presence of normal or saline environments, where 2 mM Na₂SiO₃ gave the highest increment. Silicon-induced growth stimulation may be due to high water-use efficiency, better absorption of nutrients, balance of K⁺/Na⁺ ions, maintenance of membrane stability, cell wall strengthening, activation of antioxidant systems that reduce oxidative stress caused by ROS, and others. Also, silicon has been proven to have positive effects on chloroplast integrity and photosynthesis, as well as the functionality of membranes in saline environments, maintaining the biosynthesis of biomass despite osmotic and ionic stresses [39–41]. It appears that the best results obtained for 2 mM treatments in this experiment can be explained by its ability to give the most favorable ratio of silicon supply and plant responsiveness in order to overcome salinity-induced growth inhibition.

These results are in agreement with earlier works highlighting the positive role of silicon in the growth of plants grown under salinity stress. Ahmad et al. [42] found that the addition of silicon greatly promoted maize growth and biomass accumulation under salt stress due to an improvement in plant water status and physiology. Also, Ferrández-Gómez et al. [18] showed that treatment with Na₂SiO₃ greatly alleviated salinity-induced growth inhibition by decreasing Na⁺ toxicity, maintaining ionic balance and sustaining photosynthesis. In the same way, Wang et al. [43] have found that nano-silicon greatly promoted tomato growth under salinity conditions due to improved antioxidant metabolism, reduced oxidative stress, and nutrient absorption. Thus, all the above-mentioned results are consistent with the present results, confirming that silicon, especially at the optimum dose of 2 mM Na₂SiO₃, can effectively alleviate negative effects of salinity on the growth of plants.

However, salinity stress resulted in the significant decrease in chlorophyll a, chlorophyll b, carotenoid, and total chlorophyll levels, with the effect being more pronounced at elevated NaCl levels. The above reduction is a characteristic feature of osmotic and ionic stress caused by excess salts, which leads to the deterioration of chloroplast structure, inhibition of chlorophyll synthesis, decomposition of pigments, and disturbance of the electron transport chain in photosynthesis. Also, excessive amounts of Na⁺ and Cl⁻ result in the enhanced generation of ROS, causing the lipid peroxidation of the chloroplast membrane and decomposition of photosynthetic pigments [7]. In contrast, the external addition of Na₂SiO₃ resulted in the significant restoration of the pigment levels under salt stress, with 2 mM Na₂SiO₃ showing the best results. The possible explanation for such effect of Si is its contribution to the preservation of chloroplast integrity, stabilization of thylakoid membranes, better absorption of such elements as Mg, N, and Fe needed for chlorophyll synthesis, maintenance of ionic balance through the prevention of Na⁺ accumulation, and activation of the antioxidant mechanisms of chloroplast protection [42].

These findings are consistent with previous research indicating the importance of silicon in protecting photosynthesis during salinity. Ferrández-Gómez et al. [18] found that sodium silicate treatment could restore the amount of chlorophyll and photosynthetic activity of tomato plants stressed with salt due to a reduction in Na⁺ toxicity and functioning of chloroplasts. Similarly, Gou et al. [44] observed that silicon slows down chlorophyll breakdown and leaf senescence through cytokinin synthesis and improvement of the antioxidant activity, thus preserving photosynthetic activity under salinity. The same observations were made by Irfan et al. [45] and Wang et al. [43] who suggested that the beneficial effect of silicon is provided through the increased stability of chlorophyll pigments owing to efficient ROS removal, nutrient balance, and protection of the photosynthesis apparatus. Consequently, all this data is consistent with the current research results, which indicate that the treatment with 2 mM Na₂SiO₃ prevents the negative impact of salinity on photosynthesis through maintaining chlorophyll pigments and chloroplasts functioning.

Salinity stress caused high concentrations of electrolyte leakage (EL), malondialdehyde (MDA), hydrogen peroxide (H₂O₂), and hydroxyl radical (•OH). High concentrations of these substances suggest high levels of oxidative stress and damage to membranes. Excess production of ROS under salinity causes membrane damage via lipid peroxidation. In contrast, application of Na₂SiO₃ reduced concentrations of EL, MDA, H₂O₂, and •OH, with the highest reduction noted for 2 mM Na₂SiO₃. These results demonstrate that silicon decreases oxidative stress through improving antioxidant defense and maintaining membrane stability. The obtained data are consistent with those described by Ferrández-Gómez et al. [18] about the effect of silicon on decreasing membrane permeability and lipid peroxidation in tomato plants exposed to salinity. Furthermore, Wang et al. [43] described the effects of silicon on increasing antioxidant capacity and reducing ROS levels under salinity. All these results prove the protective effect of silicon on tomato plants against oxidative stress.

It was shown in the current study that proline production was increased under salt stress conditions in a more pronounced way under severe salt stress. Proline is one of the major osmoprotectants that can regulate osmotic adjustment, maintain protein integrity, protect membranes, and detoxify ROS. Treatment of plants with Na₂SiO₃ increased proline production, especially at the 2 mM dose, and indicated that silicon can contribute to salt stress tolerance through osmotic and antioxidant protection of cells. Our results correspond with those of Hernández-Salinas et al. [40] and Aouz et al. [46], who emphasized the protecting function of proline under salt stress. Nevertheless, Ferrández-Gómez et al. [18] revealed the decrease in proline production under silicon treatment. The discrepancy can be explained by different plant genotypes, salt stress intensity, silicon doses, and conditions of the experiments.

The greater accumulation of AsA and GSH in the presence of salt stress demonstrates the activation of the non-enzymatic antioxidant defense system due to high levels of ROS generation. Both antioxidants serve as important factors that help maintain the redox homeostasis within cells via the AsA-GSH cycle. In the current research, treatment with Na₂SiO₃ stimulated the accumulation of AsA and GSH in both control and salt-stressed plants, especially at 2 mM Na₂SiO₃ concentration, which can imply that silicon can enhance the efficiency of ROS scavenging and antioxidant potential. The current results are consistent with the research by Hasanuzzaman et al. [41] in which it was shown that salinity-induced accumulation of AsA and GSH serves as an effective mechanism for the ROS scavenging and oxidative stress resistance. Khan et al. [47] and Wang et al. [48] also showed that the application of silicon enhances redox homeostasis due to the stimulation of non-enzymatic antioxidant production. The recent works of Mir et al. [49], Arif et al. [50], and Mahmood et al. [51] have also shown the significant role of stimulation of antioxidant metabolism by silicon in increasing plant salinity tolerance.

In response to salt-induced stress, the antioxidant response system is stimulated in order to protect the cells against any oxidative damage since there is a marked rise in the activity of SOD, CAT, APX, GR, and POX enzymes. From the mechanism point of view, the activity of SOD is considered to be the first enzymatic line of defense, as it dismutates quickly superoxide radicals into hydrogen peroxide and molecular oxygen. The formed hydrogen peroxide is decomposed into water and oxygen molecules by CAT enzymes in peroxisomes, as well as by APX enzymes within the ascorbate-glutathione (AsA-GSH) cycle [52–55]. Meanwhile, GR ensures maintenance of the cellular high content of reduced glutathione due to its role in NADPH-dependent reduction of oxidized glutathione. Thus, the efficiency of AsA-GSH cycle is continuously maintained in the cellular redox balance [19]. In the current study, the activity of those enzymes increased substantially due to Na₂SiO₃. These results are supported by the work of Zhu et al. [56] and Hasanuzzaman et al. [41] who reported the enhancement in the activities of major antioxidant enzymes such as SOD, CAT, APX, and GR due to the effect of silicon in decreasing the ROS-mediated damage under salinity stress. Likewise, Siddiqi et al. [57] and Wang et al. [48] have reported that the treatment of silicon on tomato plants resulted in increased antioxidant activity, decreased H₂O₂ production, and protection against the oxidative damage under salt stress conditions. Additionally, Arif et al. [50] highlighted that the activation of antioxidant enzymes through silicon is necessary to maintain redox homeostasis.

In the current experiment, stress due to salinity resulted in significant reductions in N, P, K⁺, and Si concentrations and an increase in Na⁺ concentrations, showing extreme disturbances in mineral homeostasis. Such alterations are attributed primarily to Na⁺-mediated ionic toxicity and competition for Na⁺ and essential nutrients in the processes of uptake and transport. On the contrary, treatment with Na₂SiO₃ under saline conditions led to a decrease in Na⁺ accumulation and increased acquisition of N, P, K⁺, and Si, resulting in ionic balance and better nutrient uptake. The results are consistent with previous reports, where silicon-mediated salt tolerance has been shown to be linked to limited uptake of Na⁺, Na⁺/K⁺ ratio maintenance, and mineral nutrition [18, 50, 53]. These results show directly that Si treatment increased plant growth, photosynthetic pigments, membrane integrity, water status, activities of antioxidant enzymes, and ionic homeostasis especially Na + and K+ uptake and the ratio of K+/Na+. The mechanisms like chloroplast protection, increased cytokinin biosynthesis, activation of H+-ATPase activity of plasma membrane, and Na⁺ exclusion were not directly studied in the present study. Therefore, increased levels of chlorophyll and carotenoids were explained as indication for improved maintenance of photosynthetic activity, and chloroplast structure protection was considered to be the possible mechanism. Similarly, the possible roles of cytokinin biosynthesis and H+-ATPase activity were suggested as the explanation for the observed effects as the result of previous studies [58–63].

Exogenous silicon treatment plays a crucial role in alleviating ionic toxicity caused by salt stress by controlling the expression of various transport proteins such as Na⁺ plasma membrane antiporters (SOS1), vacuole exchangers (NHX), and potassium channels (HKT). The role of silicon treatment is to increase the expression of SOS1 protein to facilitate faster efflux of toxic Na⁺ ions as well as limit their movement from roots to shoots, and at the same time, activate the NHX transporters in the tonoplast to facilitate safe accumulation of Na⁺ ions in vacuoles. In addition, silicon treatment controls the unrestricted movement of Na^+^ ions through the HKT pathway and maintains high-affinity potassium uptake (AKT1), thus ensuring a high intracellular K^+^/Na^+^ ratio [64, 65].

## Conclusion

**Fig. 10:**
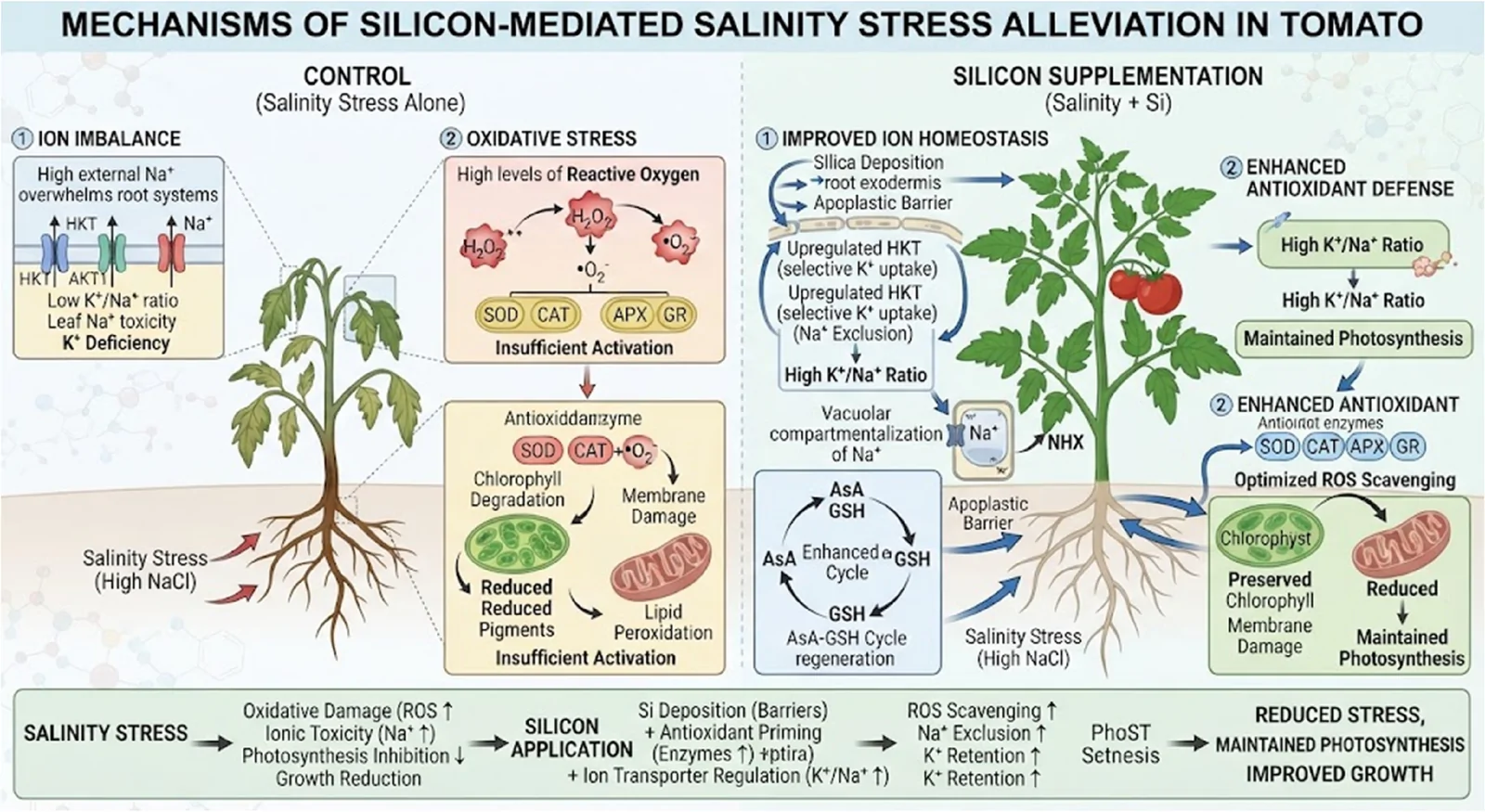
Schematic model summarizing how silicon alleviates salinity stress in tomato

The current study has shown that the use of exogenous Na₂SiO₃ is a successful tool in coping with salinity stress in tomato (Fig. 10). From among different concentrations studied, 2 mM Na₂SiO₃ was found to be the most efficient, promoting plant growth, ensuring photosynthetic capacity, and balancing ions by decreasing Na⁺ content and improving the uptake of essential nutrients. Furthermore, the addition of silicon was found to increase enzymatic and non-enzymatic antioxidant activities, reduce oxidative injury, and maintain the integrity of cellular membranes during salt stress. The results of this study reveal the effectiveness of silicon biostimulants in improving crop productivity in salt-affected soils. Nevertheless, more research under field conditions and involving various genotypes is required to prove its practical use. Further studies are expected to uncover the molecular basis of silicon-induced tolerance to salt stress.

### Limitation

A limitation of the present study is the use of Na₂SiO₃ as the silicon source, because its application simultaneously introduces additional Na⁺ into the growth system. The 1, 2, and 3 mM Si treatments supplied approximately 2, 4, and 6 mM Na⁺, respectively. Although Si-treated plants exhibited reduced Na⁺ accumulation and improved K⁺/Na⁺ ratios under salinity, the contribution of the additional Na⁺ from Na₂SiO₃ cannot be completely separated from the effects of Si under the present experimental design. Therefore, future studies should include an Na⁺-matched control and/or a sodium-free Si source to distinguish the specific effects of Si from those associated with the sodium component of the silicate formulation.

## Data Availability

The data sets generated and analyzed in this study are available from the corresponding author on reasonable request.
